# Does penile rehabilitation have a role in the treatment of erectile dysfunction following radical prostatectomy?

**DOI:** 10.12688/f1000research.12066.1

**Published:** 2017-10-31

**Authors:** Gideon Blecher, Khaled Almekaty, Odunayo Kalejaiye, Suks Minhas

**Affiliations:** 1 University College London Hospitals NHS Foundation Trust, 16-18 Westmoreland Street, London, W1G 8PH, UK; 2 Urology Department, Tanta University, Tanta, Egypt

**Keywords:** Erectile dysfunction, Radical Prostatectomy, Prostate Cancer, Penile rehabilitation, Urology

## Abstract

In men undergoing radical treatment for prostate cancer, erectile function is one of the most important health-related quality-of-life outcomes influencing patient choice in treatment. Penile rehabilitation has emerged as a therapeutic measure to prevent erectile dysfunction and expedite return of erectile function after radical prostatectomy. Penile rehabilitation involves a program designed to increase the likelihood of return to baseline-level erectile function, as opposed to treatment, which implies the therapeutic treatment of symptoms, a key component of post–radical prostatectomy management. Several pathological theories form the basis for rehabilitation, and a plethora of treatments are currently in widespread use. However, whilst there is some evidence supporting the concept of penile rehabilitation from animal studies, randomised controlled trials are contradictory in outcomes. Similarly, urological guidelines are conflicted in terms of recommendations. Furthermore, it is clear that in spite of the lack of evidence for the role of penile rehabilitation, many urologists continue to employ some form of rehabilitation in their patients after radical prostatectomy. This is a significant burden to health resources in public-funded health economies, and no effective cost-benefit analysis has been undertaken to support this practice. Thus, further research is warranted to provide both scientific and clinical evidence for this contemporary practice and the development of preventative strategies in treating erectile dysfunction after radical prostatectomy.

## Introduction

Erectile dysfunction (ED) is common after many types of urological surgery, including radical prostatectomy (RP). Given that many of these operations are performed in relatively healthy, young and middle-aged men, any subsequent impairment of erectile function (EF) may have significant long-term psychological and functional morbidity ^1^.

RP is perhaps the most common urological procedure affecting EF, although other procedures have been implicated, including Peyronie’s disease surgery, transurethral resection of the prostate, and circumcision ^2– 4^.

Prostate cancer is the most common cancer in UK males, and annually over 45,000 cases are diagnosed, whilst over 11,000 men die from this disease ^5^. In recent years, many efforts have been made to minimise the detrimental impact of prostate cancer treatment on sexual function. Whilst there are limited randomised controlled studies in strong support of penile rehabilitation, various strategies have been adopted as preventative measures to reduce the risks of surgery on EF; the mainstays of treatment include oral agents (phosphodiesterase inhibitors), vacuum pump devices, and intracavernosal injections. Other novel treatments, along with the current understanding and advances in the management of ED following RP, will also be discussed in this commentary.

## Pathophysiology of erectile dysfunction after radical prostatectomy

Penile erection is a neurovascular event requiring intact neural and vascular pathways. The pro-erectile neural pathways are derived from the spinal cord segments S2–S4. The pelvic plexus from which the cavernous nerves take their origin innervates the penis. Nitric oxide is produced both from the endothelium lining the corporal sinusoidal spaces and from non-adrenergic non-cholinergic nerves by the enzyme nitric oxide synthase (NOS), which leads to corporal smooth muscle relaxation ^6^. The resultant reduction in intra-cellular calcium levels leads to corpus cavernosal smooth muscle relaxation and initiation of the veno-occlusive mechanism and penile erection ^7^. Stimulation of the sympathetic nerves leads to corporal smooth muscle contraction.

The neurovascular bundle, containing the erectile nerves, courses along the anterolateral aspect of the prostate ^8^. Damage to the cavernous nerves, arterial blood supply, cavernosal disease or veno-occlusive dysfunction may result in ED.

RP can be performed in either open or minimally invasive methods, the latter either purely laparoscopically or with robotic assistance. During RP, mechanical and thermal injury may occur to the neurovascular bundle during the lateral or apical dissection or during excision of the seminal vesicles. Ligation of accessory pudendal/obturator arteries may further impair EF. Other theories for ED after RP include local inflammatory effects, trabecular smooth muscle ischaemia with subsequent apoptosis (leading to a reduction in smooth muscle–to–collagen ratio), and veno-occlusive dysfunction ^9– 12^.

**Figure 1.**
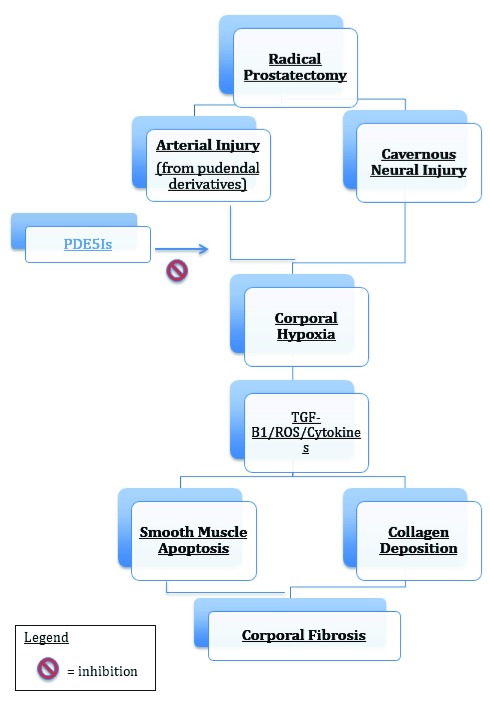
Pathophysiology of erectile dysfunction after radical prostatectomy. ROS, reactive oxygen species; TGF-β1, transforming growth factor beta 1.

The resultant ED appears to result from progressive corporal smooth muscle fibrosis combined with a loss of elasticity ^13^. This process appears to be mediated by various cytokines, including transforming growth factor beta 1, endothelin 1, human tissue kallikrein 1, and reactive oxygen species as well as the Rho-kinase/LIM-kinase/cofilin pathway ^14– 16^. In animal models in which the cavernous nerves are injured, there is a reduction in the expression in NOS in both the nerves and endothelium of the penis with an increase in expression of endothelin 1 ^17^. A number of studies support the concept that phosphodiesterase type 5 inhibitors ****(PDE5Is), via their ability to increase cGMP levels, are able to inhibit collagen synthesis by imparting an anti-fibrotic and anti-apoptotic effect ^18, 19^ ( Figure 1).

Two studies in humans, without controls, have examined the histological changes associated with PDE5Is ^20, 21^. In one study ^20^, 21 patients had biopsies of cavernosal muscle following treatment with either 50 or 100 mg of sildenafil for 6 months following prostatectomy. The 100 mg dose arm led to an increase in smooth muscle content from 43% to 57%, but there was no difference for the 50 mg group. The study by Iacono *et al*. ^21^ examined 21 patients who received 50 mg sildenafil three times per week. There was no change in elastic or connective tissue content.

Predictive factors for ED after RP appear to be related to pre-operative baseline function and patient age and whether (and to what degree) a cavernous nerve-sparing procedure has taken place ^22, 23^. In a prospective study of 314 men, men younger than 60 years of age (compared with those older than 65) had EF recovery 3 years after RP of 76% versus 47%, respectively ^23^. The same study demonstrated that full pre-operative potency, versus men with recently diminished EF versus those with impaired erections, had potency recovery rates of 54%, 37% and 22%, respectively. A bilateral versus unilateral versus non–nerve-sparing approach also significantly affected potency rates: 55%, 41% and 21%, respectively.

## Evidence of erectile dysfunction after radical prostatectomy

Advances in our understanding of the anatomy of the neurovascular bundles with advances in surgical technique as described by Walsh *et al*. ^24^ (that is, the anatomical nerve-sparing RP) have enabled a reduction in the risks of post-operative ED ^25^. However, the challenge for the urological oncologist is to maintain post-operative EF by sparing the cavernous nerves whilst ensuring negative oncological margins. This concept becomes more complicated as the general trend to monitor men with low-risk disease has become more mainstream. In particular, historical studies reporting on the outcomes from RP included those patients with low-risk disease in whom preservation of the neurovascular bundle and hence maintenance of EF was more likely. It is now apparent that a disproportionate number of men with low to intermediate risk have undergone such surgery and as such the reported rates of EF are probably higher than those in more contemporary series. Therefore, it is predicted that the proportion of men with high-risk disease (prostate-specific antigen of more than 20 ng/dL, bilateral disease (pT2c) or greater, and Gleason grade of 8–10) undergoing RP will increase, with a potential rise in ED ^26^. However, nerve sparing in high-risk patients is being pursued ^27^. Thus, will advances in surgical technique ensure good functional outcomes in higher-risk disease, or will we see a potential reduction in the number of nerve-sparing procedures performed and therefore a greater proportion of men with ED?

Regardless of the answers to these questions, the starting point for analysing data on penile rehabilitation is the definition of ED. Unfortunately, there remains significant heterogeneity in the literature in terms of definitions of ED after RP, and a significant number of studies do not clearly state their definitions of ED or return to sexual function. Furthermore, clarity on whether adjunctive treatments such as PDE5Is are used is not uniform. Scoring systems such as Sexual Health Inventory For Men (SHIM) scores, International Index of Erectile Function (IIEF-5), sexual questionnaires, and patient and partner reporting are all prone to inaccuracies, and comparative indices such as return to baseline function are not used.

A meta-analysis in 2009 noted that the ‘overall rate’ of ED after RP was 58%; however, within the 22 included studies, there existed 22 different definitions of EF outcome ^28^. Moreover, reporting on EF after RP is further clouded by variability in the timing of pre-operative assessment of EF, which can significantly affect the ‘accuracy’ of scores; one can imagine that a pre-operative patient who has a recent cancer diagnosis may have fewer sexual thoughts and thus potentially reduced perception of their EF compared with that prior to their diagnosis ^29^. As such, defining and reporting the rate of ED after RP are fraught with problems. Rather than scoring on validated scoring systems (for example, IIEF-5, SHIM or Global Assessment Questionnaire), a ‘return to baseline’ assessment may provide a more realistic measure and comparator of health-related quality-of-life outcomes in this group of patients.

Furthermore, studies often do not report on the effects of multi-modal treatments; that is, patients may receive adjuvant treatments, which may affect EF, including radiotherapy or androgen deprivation. These issues aside, evidence from the literature would suggest that ** the probabilities of EF (measured as IIEF-6 score of at least 22) at 24, 36 and 48 months were 22%, 32% and 40%, respectively ^30^, and whilst erectile recovery occurred up to 48 months post-operatively, EF appears to remain stable thereafter ^31^.

## Rehabilitation techniques

### Phosphodiesterase type 5 inhibitors

The introduction of PDE5Is has revolutionised the management of ED. Various pharmacotherapies have been used for penile rehabilitation after RP. However, there are only a limited number of randomised trials exploring the efficacy of PDE5Is in this setting. Whilst trial subjects have had nerve-sparing RP, the exact extent of nerve spare, surgeon volume, or experience is not universally presented and analysed. As discussed previously, the exact mechanism of how PDE5Is may improve long-term EF rates remains unclear, although animal studies indicate a possible neuroprotective effect ^32, 33^.

PDE5Is, including sildenafil, tadalafil, avafanil and vardenafil, are oral agents which can be administered as an on-demand treatment for ED. In the non-RP population, there is evidence that low-dose daily regimens may provide better IIEF outcomes compared with the on-demand approach ^34^. There is no clear answer in the RP population regarding daily versus on-demand dosing but several trials attempt to address this question ^35– 39^ ( Table 1).

**Table 1.** Randomised trials of oral phosphodiesterase type 5 inhibitors.

Authors	Year	Subjects	Treatment	Measure	Duration	Comment
Brock *et al*. ^40^	2003	440	Placebo versus vardenafil 10 mg, 20 mg	IIEF-EF Diary questions Global Assessment Question	3 months	Benefit in severe erectile dysfunction
Montorsi *et al*. ^35^	2008	628	Placebo versus vardenafil (5 mg to 20 mg); nightly versus on-demand	IIEF	9 months, 2-month washout	On-demand better than nightly, better than placebo
Padma-Nathan *et al*. ^41^	2008	76	Placebo versus nightly sildenafil 50 mg, 100 mg	IIEF	36 weeks, 8-week washout	Premature closing due to lack of treatment effect. Results after 12 months of follow-up; 4% of placebo patients regained spontaneous erection compared with 27% in treatment arm
Mulhall *et al*. ^42^	2013	298	Avanafil on-demand versus placebo	Sexual encounter profile (question 2) Sexual intercourse IIEF-EF	3 months	
Pavlovich *et al*. ^36^	2013	100	Sildenafil; on-demand with nightly placebo versus nightly with on-demand placebo	Quality-of-life assessments	12 months, 1-month washout	No significant differences found. No sole placebo group
Montorsi *et al*. ^37^	2014	423	Placebo versus tadalafil; daily versus on-demand	IIEF-EF Sexual encounter profile Penile length	9 months, 6-week washout, 3-month open- label	Penile length loss reduced by 4.1 mm Unassisted erectile function not improved after treatment cessation. No benefit to tadalafil versus placebo
Moncada *et al*. ^38^	2015	423	Placebo versus tadalafil; daily versus on-demand	IIEF-EF	9 months, 6-week washout	IIEF-EF improved most with daily (29.5%, 23.9%,18.4% with IIEF ≥22)
Kim *et al*. ^39^	2016	74	On-demand sildenafil with addition of either nightly sildenafil or placebo	Rigiscan/IIEF	12 months, 1-month washout	No benefit of additional nightly dosing to on-demand. No pure placebo group

IIEF, International Index of Erectile Function; IIEF-EF, International Index of Erectile Function–erectile function domain.

In patients who have undergone RP, the earliest randomised trial, by Brock *et al*. in 2003, was a placebo-controlled prospective trial examining the use of vardenafil (both 10 and 20 mg doses) with assessments at 3 months ^40^. The authors report an improvement for both dosages, in IIEF-EF, global assessment and diary questions, but only for men with severe ED (IIEF < 11). Limitations included a short follow-up period.

Montorsi *et al*. evaluated 628 patients for 9 months in the multi-centre REINVENT randomised trial ^35^. The double-blind placebo-controlled trial with a 2-month washout period, compared the efficacy of daily 5 mg tadalfil versus 20 mg on demand. On-demand use of vardenafil was superior to both nightly dosing and placebo, and the proportions of patients with IIEF-EF scores of at least 22 were 48%, 32% and 25%, respectively. However, following the washout period, no statistically significant differences between the treatment arms were noted.

In the same year, Padma-Nathan *et al*. prematurely ceased their enrolment of patients in a comparative study of nightly sildenafil versus placebo because of a lack of treatment efficacy ^41^. This three-phase trial was structured such that results were observed 12 months following surgery. The authors suggested that 4% of placebo group versus 27% of the sildenafil group described adequate EF following the 8-week washout period ^41^. The authors conclude that, despite not meeting their initial power requirements, nightly sildenafil can improve spontaneous erection rates compared with placebo. (Erection was defined as a combined score of at least 8 for questions 3 and 4 of the IIEF and also an answer of ‘Yes’ to the question ‘Over the past 4 weeks, have your erections been good enough for satisfactory sexual activity?’)

In a study of 298 patients, Mulhall *et al*. demonstrated a significant benefit of on-demand avanafil with 6.4% of sexual attempts (Sexual Encounter Profile question 3, or SEP3) at 15 minutes or less as successful compared with 4.5% for placebo ^42^. A short duration of follow-up (3 months) and failure to measure EF after a washout period are criticisms of this trial.

In contrast, the study by Pavlovich *et al*. showed no differences in EF after 12 months; their design, however, did not include an adequate placebo group ^36^. A larger study by Montorsi *et al*. also failed to show a benefit after treatment cessation ^37^. Fifty centres participated in this multi-centre trial, in which 423 patients were randomly assigned to placebo or tadalafil and which had both on-demand 20 mg and nightly 5 mg arms. However, there was a reduction in loss of penile length in the daily tadalafil group: 2.2 mm in the daily compared with 7.9 mm in on-demand and 6.3 mm in placebo groups.

The most recent trial, of 74 patients by Kim *et al*., examined both subjective assessment of EF as well as nocturnal penile tumescence with Rigiscan (GoTop Medical, Saint Paul, MN, USA) ^39^. After 12-month treatment with on-demand sildenafil (100 mg), patients received either nightly sildenafil or placebo. Despite a unique method of EF assessment, there was no pure-placebo arm. Their results indicated no benefit of nightly dosing compared with on-demand treatment.

Thus, overall, the literature on the role of PDE5Is in the context of penile rehabilitation is contradictory. It is interesting that despite the absence of definitive clinical evidence for penile rehabilitation, clinicians often use PDE5Is for penile rehabilitation ^43^, but questions remain as to the efficacy, duration and cost utility of such treatment.

### Intracavernosal injections

Intracavernosal injections are used as second-line agents for the treatment of ED, most commonly alprostadil, which is prostaglandin E _1_ (PGE _1_). PGE _1_ lowers intra-cellular calcium via cyclic GMP, leading to smooth muscle relaxation. Other agents include phentolamine (alpha antagonist), papaverine (increases cAMP) and aviptadil. The major disadvantage is pain associated with the injection ^44^, whilst the benefit is pharmacological efficacy despite a non–nerve-sparing procedure. There are only a few randomised prospective studies assessing the role of intracavernosal injections as a form of penile rehabilitation. The randomised trial by Montorsi *et al*. 20 years ago, though of relatively short follow-up and small numbers (n = 30), demonstrated an improved EF rate with three weekly injections of intracavernosal alprostadil ^45^. Although the trial had no placebo arm and was non-blinded, a further 132 patients were randomly assigned to oral sildenafil with and without intracavernosal alprostadil three times a week in a study by Mulhall *et al*. ^46^. This study demonstrated that after 18 months, use of alprostadil led to a spontaneous erection rate of 53% versus 19% for those taking sildenafil only ^46^. A small (n = 22) non-placebo study by Nandipati *et al*. ^47^ used either intracavernosal alprostadil or Trimix (PGE _1_, papaverine and phentolamine) with oral sildenafil versus sildenafil alone. Eleven out of 22 (50%) reported return of spontaneous erections at a mean of 6 months but none sufficient for intercourse. With treatment, sildenafil alone did not improve the IIEF compared with baseline post-operatively, whereas injections or combination injections with sildenafil did improve the IIEF. All four subjects using Trimix achieved erections sufficient for intercourse without sildenafil. Importantly, these results are not ‘unaided’ IIEF scores and it is not clear that study participants were blinded to the treatments. Although these studies form the basis of our current understanding, they are limited by small numbers and lack of placebo control, and some are non-randomised, and results do not include return to baseline figures—an important measure of EF after RP.

### Intraurethral alprostadil

Intraurethral alprostadil is available as a suppository, although both urethral discomfort and questionable efficacy are factors which may influence clinician prescribing. This treatment is often plagued by high dropout rates. The International Consultation for Sexual Medicine (ICSM) recommendations ^48^ suggest that it has a role in the management of ED after RP. There is only one trial assessing its role in the context of penile rehabilitation after RP ^49^. Raina *et al*. conducted a randomised, placebo-controlled, prospective trial of 91 patients who received three doses per week for six months ^49^. Seventy-four percent in the active arm versus 37% regained erections sufficient for intercourse; however, most men (71%) who regained their EF were dissatisfied and sought alternative treatments. A high dropout rate of 32% was noted. No intention-to-treat analysis was performed, thus tainting the analysis. A randomised prospective trial by McCullough *et al*. investigated intra-urethral alprostadil versus oral sildenafil ^50^. One hundred thirty-nine men were assessed following 9 months of treatment; no statistical difference was found in IIEF or intercourse success rates ^50^.

Alprostadil is also available as a topical application; however, no prospective randomised trials in relation to penile rehabilitation are currently available ( Table 2 and Table 3).

**Table 2.** Randomised trials of intracavernosal alprostadil.

Authors	Year	Subjects	Treatment	Measure	Follow-up	Comments
Montorsi *et al*. ^45^	1997	30	IC alprostadil versus placebo	Sexual history Doppler US Nocturnal polisomnograph	6 months	67% versus 20% recovery of erectile function Non-blinded
Mulhall *et al*. ^46^	2005	132	Sildenafil versus sildenafil + IC alprostadil	IIEF	18 months	53% versus 19% spontaneous erections. Higher IIEF with treatment
Nandipati *et al*. ^47^	2006	22	IC alprostadil or Trimix + sildenafil versus sildenafil	SHIM Penile Doppler	12 months	Small study. No placebo. Not blinded.

IC, intracavernosal; IIEF, International Index of Erectile Function; SHIM, Sexual Health Inventory For Men.

**Table 3.** Randomised trials of vacuum erection device.

Authors	Year	Subjects	Treatment	Measure	Follow-up	Comments
Engel ^55^	2011	23	Tadalafil three times per week with VED versus tadalafil alone	IIEF Penile Hardness Scale Sexual questionnaire	12 months	Low patient numbers
Raina *et al*. ^54^	2006	109	VED versus no treatment	SHIM Compliance Change in penile length Return of natural erection Intercourse ability	9 months	17% had erections sufficient for intercourse with VED versus 11% 18% discontinued treatment

IIEF, International Index of Erectile Function; SHIM, Sexual Health Inventory For Men; VED, vacuum erection device.

### Vacuum erection device

The vacuum erection device (VED) is a recognised therapeutic option for managing ED; however, data relating to its role in rehabilitation after RP are limited. VED increases glanular and corporal oximetry ^51^, and animal studies suggest that the mechanism of action may relate to the anti-hypoxic, anti-apoptotic and anti-fibrotic effects via reduced hypoxia-inducible factor 1-alpha and transforming growth factor beta 1 ^52^. A small pilot study in 2007 randomly assigned 28 men to a VED daily for 5 months, for 10 minutes a day, versus no treatment. At 6 months, IIEF scores were higher in the VED group: 12.4 versus 3.0 ^53^. Raina *et al*. analysed 109 patients who received VEDs versus control (no erectogenic treatment) for 9 months after prostatectomy ^54^. Return of natural erections was reported in 32% in the VED group and in 37% of the controls. Though lacking a control group, Engel compared 23 men taking tadalafil versus tadalafil plus VED for 12 months ^55^. IIEF was higher for the combination group at 6, 9 and 12 months, as was ability to penetrate vaginally. However, the low power of this study is a major limitation. Importantly, these studies are weakened by selection bias and lack of blinding and randomisation.

## Timing of rehabilitation

The majority of studies start treatment within the first four weeks post-operatively. Mulhall *et al*. ^56^ performed a case review of 84 patients who received PDE5Is and intracavernosal alprostadil (on-demand) within or after 6 months after RP. There was a difference of IIEF EF scores: 22 versus 16 in favour of early treatment; however, this level 4 evidence article is potentially exposed to selection bias. No prospective randomised trials are available to answer this contentious issue.

The duration of rehabilitation is another unanswered question. Yiou *et al*. retrospectively assessed 75 patients who received intracavernosal alprostadil for 24 months; only those patients who did not respond to PDE5I at 12 months continued injection treatment ^57^. No significant improvement in spontaneous erections was noted between 12 and 24 months. Thus, although practitioners may suggest treatment for variable durations, no evidence exists to counsel patients on this important aspect of pharmacotherapy.

## Novel therapies - clinical

### Penile vibratory stimulation

This technique has been employed for patients with and without spinal cord injury to achieve erection but also for ejaculation. There exist a number of devices, including the Vibererect device (Reflexonic, Chambersburg, PA, USA) and Ferticare vibrator (Multicept, Frederiksberg, Denmark), that stimulate the pudendal nerve to causes a reflex parasympathetic response, resulting in erection. Fode *et al*. performed a randomised prospective controlled trial of penile vibratory stimulation versus control in 68 patients following nerve-sparing RP ^58^. At 12 months, there was a trend towards improved IIEF-5 scores (median of 18 versus 7.5); however, this result did not reach statistical significance between treatments ^58^. Vibratory stimulators are not currently recommended for routine treatment of ED by either the European Association of Urology (EAU) Guidelines or the ICSM.

## Novel therapies - preclinical

### Sonic Hedgehog protein

This protein is a signal pathway protein involved in organ development ^59– 61^. Studies have analysed its role in neural regeneration. It appears that application of Sonic Hedgehog (SHH) in the immediate period of nerve damage (in rat models) may positively affect neural regeneration ^62^. In a recent study in a rat model with bilateral cavernous nerve injury, nanofiber hydrogel was used to deliver SHH to the injury site. SHH was shown to prevent neuronal and surrounding supportive glial cell degeneration ^63^ and thus may be of value in the setting of prostatectomy and ED prevention.

### Gene therapy

A variety of options are being explored in rat models for gene therapy in ED. This requires injection of either viral or non-viral vector, containing the specific therapy ^64^. Examples include upregulation of NOS (endothelial NOS) ^65^, growth factors (for example, neurotrophin-3 ^66^, glial cell–derived neurotrophic factor ^67^, brain-derived neurotrophic factor ^67^, and vascular endothelial derived growth factor ^68^), or modulation of potassium channels via Maxi-K or BK ^69^. Rat model trials have demonstrated a prevention of age-related decrease in intra-corporal pressures following cavernous nerve stimulation after an injection of pcDNA/ *hSlo,* which increases the expression of Maxi-K channels ^70^.

COX-2-10aa-PGIS is a protein involved in the production of prostacyclin, a potent smooth muscle relaxant, and has been investigated in rat models ^71^. Rats undergoing COX-2-10aa-PGIS gene therapy demonstrated improved EF as measured by intracavernosal pressure following bilateral cavernous nerve crush. In a similar attempt to increase intracavernosal prostacyclin, transfection of SuperEnzyme (a recently engineered protein) may be a potential option for gene therapy in penile rehabilitation ^72^.

### Stem cell therapy

Stem cell treatment is often looked upon as a holy grail of future medical treatments. In 2004, a study in rat models investigated injection of neural embryonic stem cells into the pelvic ganglia; those with the injections had significantly higher intracavernosal pressures ^73^. There are over 20 other studies of mesenchymal stem cell injections in rat models, and there was improvement in EF in many of these ^74^. A human trial was performed by Yiou *et al*. whereby 12 patients with localised prostate cancer were injected autologous bone marrow mononuclear cells ^75^. After 6 months, the treatment group showed significantly improved IIEF-EF (17.4 ± 8.9 versus 7.3 ± 4.5) and erection hardness (2.6 ± 1.1 versus 1.3 ± 0.8) scores. The authors rightly note that these are early data and need further phase 2 clinical trials.

## Penile implants

Though an end-stage treatment for ED rather than a rehabilitation tool, penile implant is worth mentioning. There exist both three-piece inflatable as well as malleable devices to provide support and rigidity for penetrative intercourse. For inflatable devices, a reservoir is placed in retroperitoneal, retropubic or ectopic positions. Unfortunately, complicating issues such as floppy glans, lack of glans engorgement, and changes in sensation will never enable this option to fully replicate natural erections and clearly it is a non-reversible step. Thus, though a functional end-stage option for ED, it cannot be part of the armamentarium of short-term treatment to restore baseline EF.

## Guidelines

Whilst there are several randomised studies on the subject of penile rehabilitation, much of the data outcomes are complicated by conflicting results and relatively short follow-up. In this context, what do international guidelines currently recommend in terms of penile rehabilitation? The grade A EAU recommendation states that ‘pro-erectile treatments have to be given at the earliest opportunity after RP’ ^76^. This is seemingly in relation to treating ED; however, there is no explicit statement in relation to rehabilitation. Of note, the ICSM recommendations state that ‘there are conflicting data as to whether penile rehabilitation with phosphodiesterase type 5 inhibitors improves recovery of spontaneous erections’ (level of evidence 1, grade A). The American Urology Association do not have a clear statement in relation to rehabilitation other than that ‘The applicability of PDE5 inhibitors after RP needs to be characterized’ ^77^. Thus, even contemporary guideline recommendations seem conflicting but indicate a need for further studies.

In this context, a cost-benefit assessment is important to consider given the widespread use of penile rehabilitation. The benefit of rehabilitation—that is, ability to subsequently achieve unassisted erection or return to baseline function—seems unclear.

## Cost analysis

It can be projected that the cost of treatment of ED related to RP will increase. Prostate cancer is the commonest cancer in UK males, and over 45,000 cases are diagnosed each year; this is projected to increase to over 75,000 in 2035 ^78^. With almost one tenth of these patients undergoing major surgery for their cancer ^79^ and 56% of these men undergoing bilateral nerve-sparing and up to 66% non–nerve-sparing operations ^80^, the overall number of men potentially requiring ED treatment or rehabilitation after RP is 4,500 per year in the UK. Over a period of years, this number (cumulative incidence) becomes much more significant and almost rivals the number of UK men being prescribed PDE5Is for ED not related to prostate surgery ^81^. Given that more patients with high-risk disease will undergo radical (and likely non–nerve-sparing) surgery, there will be an increase in the number of men who receive PDE5is, which will not be efficacious or cost-effective.

## Conclusions

Penile rehabilitation has been widely adopted despite a lack of high-level evidence indicating a definitive and long-term benefit. Several studies do exist, but these currently seem conflicting and have methodological limitations. Current treatments include oral PDE5Is, intracavernosal topical or intra-urethral alprostadil, and vacuum erectile devices, whilst experimental treatments are under development. Penile rehabilitation is a significant burden to health resources in public-funded health economies, and no effective cost-benefit analysis has been undertaken to support this practice. Thus, further research is warranted to provide both scientific and clinical evidence for this contemporary practice and the development of preventative strategies in treating ED after RP.
